# Studies on the mechanical stretchability of transparent conductive film based on graphene-metal nanowire structures

**DOI:** 10.1186/s11671-015-0748-z

**Published:** 2015-01-31

**Authors:** Mi-Sun Lee, Joohee Kim, Jihun Park, Jang-Ung Park

**Affiliations:** School of Materials Science and Engineering, Wearable Electronics Research Group, Low-Dimensional Carbon Materials Research Center, Ulsan National Institute of Science and Technology (UNIST), Ulsan, 689-798 Republic of Korea

**Keywords:** Transparent electrodes, Graphene, Nanowire, Flexible electronics, Stretchable electronics

## Abstract

Transparent electrodes with superior flexibility and stretchability as well as good electrical and optical properties are required for applications in wearable electronics with comfort designs and high performances. Here, we present hybrid nanostructures as stretchable and transparent electrodes based on graphene and networks of metal nanowires, and investigate their optical, electrical, and mechanical properties. High electrical and optical characteristics, superb bendability (folded in half), excellent stretchability (10,000 times in stretching cycles with 100% in tensile strain toward a uniaxial direction and 30% in tensile strain toward a multi-axial direction), strong robustness against electrical breakdown and thermal oxidation were obtained through comprehensive study. We believe that these results suggest a substantial promise application in future electronics.

## Background

Transparent conductive films have been widely used in electronics such as touch screen panels, electrochromic smart windows, and displays. In addition, stretchable electronics have attracted tremendous recent attention due to their potential advantages including outstanding suitability on a nonplanar surface and excellent portability. Therefore, encoding stretchability into transparent electrodes has been actively explored as the key to emerge flexible optoelectronic devices. Indium tin oxide (ITO) is the most commonly used material in transparent electrodes. However, it has several critical disadvantages, such as (i) the increasing costs for indium, (ii) need for a complex vacuum process, and (iii) its mechanical brittleness under external stress. These weak points of ITO limit its use for flexible electronics. Thus, there is a clear and urgent need for new transparent conductive materials with superb mechanical properties. Various alternative candidates for ITO have been intensively pursued, including carbon nanotubes [1-6], conductive polymers [1], graphene [7-14], nanowires [2,15-22], and metal mesh structures [13,21,23-25]. A powerful candidate for a transparent electrode material comes from the random networks of metal nanowires (mNWs) [17,20,22,26,27]. First, mNWs are normally formed as networks by using various solution coating processes [28-30], which can facilitate low-cost simple fabrication of transparent electrodes. In the case of mNWs, charge transport occurs longitudinally along the NWs, and open spaces between NWs provide optical transparency. Typically, sheet resistance (*R*_*s*_) of the randomly distributed mNW networks (lower than approximately 80 Ω sq^−1^) [17,20,26] with high transmittance (higher than approximately 90%) are lower than the *R*_*s*_ of undoped, chemical vapor deposition (CVD)-grown graphene (higher than approximately 1 kΩ sq^−1^) [7,9-11] and comparable with that of ITO (lower than approximately 80 Ω sq^−1^ with transmittance of approximately 90% at 550 nm) [13]. In addition to their outstanding electrical and optical properties, the mesh-type geometries of mNWs also present superb mechanical robustness under external strains, such as bending strain (approximately 1%) [20-26] or stretching strain (approximately 50%) [22,27]. Several negatives of the mNW networks include (i) high NW-NW junction resistances, (ii) high contact resistance between the mNW networks and active materials, (iii) low breakdown voltages, (iv) poor adhesion of mNWs to flexible and plastic substrates, and (v) oxidation in harsh environments. These factors have limited their integration as transparent electrodes in commercial optoelectronic devices [17,18,20-22,27].

Another promising alternative to ITO comes in the form of graphene that can be deformed up to a strain of approximately 4% with negligible cracking [12] as well as absorbing only approximately 2.3% of visible light [14]. In addition, the theoretical minimum *R*_*s*_ of pristine graphene has been estimated to be as low as approximately 30 Ω sq^−1^ [31], but the experimental *R*_*s*_ of undoped, synthesized graphene by using various methods [7,8,10,11,32] is substantially higher than the *R*_*s*_ of ITO. Although chemical doping processes [12,33] can further reduce *R*_*s*_ of the CVD-grown graphene by increasing carrier density, the instability of the interactions between graphene and dopant typically limits the lifetime of this doping effect and leads to a time-variant increase of *R*_*s*_ [13].

In this paper, we report graphene-mNW hybrid nanostructures as high-performance, stretchable, and transparent electrodes. Especially, we focused on the study of the mechanical flexibility of the hybrid film, so various flexible tests such as bending, folding, and uniaxial and multi-axial directional stretching tests were performed. These fabricated hybrid structures present superb mechanical flexibility (folding with bending radius of approximately 3.7 μm) and stretchability (10,000 times in a fatigue test with maximum stretching strain of 100% and stretching in tensile strain of 30% toward multi-axial direction).

### Experimental methods

Silver nanowires (AgNWs) with average length of 30 μm ± 7 μm and average diameter of 20 nm ± 5 nm dispersed in ethanol (3 mg mL^−1^) were purchased from Nanopyxis Co., Ltd (Cheonan, South Korea). The solution was stored at 5 °C, and then spin-coated on substrates. The spin coating condition of AgNWs was fixed at 500 rpm for 30 s, except for the density study of AgNWs, which was done according to the spin rates. After spinning the AgNW suspension on substrates (with or without graphene as a top layer), the samples were annealed at 150 °C for 90 s to evaporate the solvent completely.

The synthesis and transfer of graphene were carried out under the following conditions. A Cu foil (Alfa Aesar, item no. 13382; Ward Hill, MA, USA) was loaded onto the center of a quartz CVD reactor under low vacuum (100 mTorr). Subsequently, the reactor was heated up to 1,000 °C with introduction of the flow of Ar (200 sccm) and H_2_ (500 sccm). The CVD growth was carried out under CH_4_ (12 sccm) and H_2_ (500 sccm) flows for 5 min, and then the reactor was rapidly cooled to room temperature under Ar (500 sccm) flow. To transfer the synthesized graphene, we used a 200-nm-thick poly(methyl methacrylate) (Micro Chem Corp. 950 PMMA; Newton, MA, USA) film as a supporting layer which was spun on the graphene. The Cu foil was dissolved in a diluted etching solution of FeCl_3_:HCl:H_2_O (1:1:20 vol.%) with the PMMA/graphene layer floating on the surface of the solution. Subsequently, the sample was rinsed by floating it on the surface of deionized water (DI water) and then the sample was transferred onto the desired substrates. In addition, the PMMA supporting layer was removed with acetone.

We have employed two approaches to assemble the hybrid structures (i) AgNWs on graphene or (ii) graphene on AgNWs: (i) After the CVD synthesis of the graphene layer, the graphene was transferred onto a target substrate. After spin casting of the AgNW dispersions onto the graphene surface, this sample was annealed at 150 °C for 90 s. (ii) AgNW network films were coated on substrate, as described above. Then the synthesized graphene layer was transferred onto the films by using PMMA. Subsequently, the supporting layer was removed with acetone.

The graphene-AgNW hybrid electrodes can be photolithographically patterned by using an etch-back process. First, the hybrid films were dry-etched using oxygen plasma (50 W, 60 sccm, 160 s) to remove carbon-based materials. Because the residues of the AgNWs were oxidized and disconnected by oxygen plasma exposure, the exposed areas became electrically nonconductive. Subsequently, these AgNW residues can be completely eliminated by using an etching solution of H_3_PO_4_:C_2_H_4_O_2_:C_6_H_4_NO_5_SNa:H_2_O (55:1:4:40 vol.%).

The preparation of a 2-μm-thick polyimide substrate for the bending test was carried out by the following procedures. After casting the 200-nm-thick PMMA sacrificial layer on a bare Si wafer, a polyimide precursor (poly(pyromellitic dianhydride-co-4,4′oxydianiline); Aldrich, St. Louis, MO, USA) was subsequently spin-coated at 3,000 rpm for 30 s. The thermal curing was processed at 250 °C for 6 h. After fabricating graphene-AgNW hybrid electrodes on the prepared polyimide surface, this 2-μm-thick polyimide film together with the hybrid electrodes could be delaminated from the bare Si wafer by eliminating the PMMA layer using acetone.

For the electrical, optical, and mechanical characterization of the prepared samples, a four-point probe method was used to measure the *R*_*s*_ using a probe station with a Keithley 4200-SCS semiconductor parametric analyzer (Keithley Instruments Inc, Solon, OH, USA). In addition, the two-point probe method was also used for the *R* measurements and electrical breakdown test using a Keithley 2425 source meter. *R*_*s*_ can be acquired by calculating it from the measured *R* by considering the aspect ratio of patterns (*R*_*s*_ = *R* × (Pattern width)/(Pattern length)). Furthermore, the change in *R* of hybrid films during uniaxial and multi-axial stretching was measured after fixing the sample on home-built stretching stages.

The optical film transmittance was assessed by ultraviolet–visible near-infrared (UV–vis-NIR) spectroscopy (Cary 5000 UV–vis-NIR, Agilent Technologies Inc, Santa Clara, CA, USA). The transmittance of the substrates was excluded in all our data. The optical images were taken using an optical microscope (BX 53, Olympus, Tokyo, Japan). The morphologies of the fabricated films were characterized using field emission scanning electron microcopy (FE-SEM) (S-4800, Hitachi, Seoul, South Korea) and atomic force microscope (AFM) (D3100, Veeco, Plainview, NY, USA).

## Discussion

### Electrical and optical properties of hybrid films

**Figure 1:**
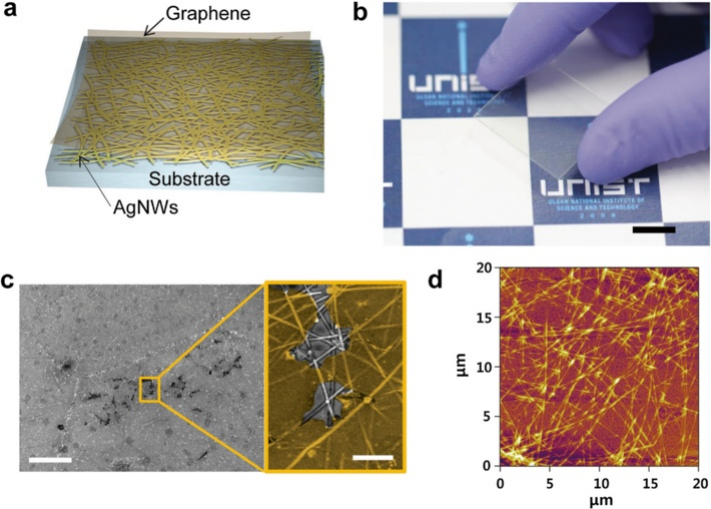
**Fabrication of graphene-AgNW hybrid film. (a)** A schematic illustration of a hybrid structure which was composed of graphene as a top layer and AgNW networks as a bottom layer. **(b)** A photograph of graphene-AgNW hybrid film fabricated on a PET. The scale bar is 1 cm. **(c)** A SEM image of torn graphene as a top layer and AgNW networks as a bottom layer. The scale bar is 10 μm (right inset: 1 μm). **(d)** An AFM image of percolated AgNW networks on graphene layer.

**Figure 2:**
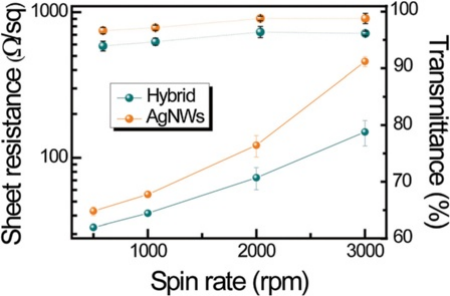
**Electrical and optical properties of hybrid films.** Plots of the sheet resistances (log scale) and optical transmittance (at the wavelength of 550 nm) of the hybrid and AgNW films according to spin rate of AgNWs.

Unlike the previous reports of related hybrid electrodes [34-36], our approach focused on mNWs with high densities above the percolation threshold. Here, above the percolation threshold means that the formation of at least one conducting pathway occurred. Moreover, this percolation threshold can be modulated by size, dimension, and number density of conducting nanomaterials [37-39]. Therefore, the percolating networks of mNWs were integrated into the graphene layer, without considerable reduction of transmittance. Here, both independently conducting components of graphene and the mNW percolating networks allow simultaneous charge transport in the hybrid nanostructures, thereby complementing the disadvantages of the other component. The transparent hybrid electrodes composed of CVD-grown graphene layer and AgNW networks were prepared by using spin coating and wet transfer methods as described in the experimental methods [40]. As shown in the schematic image of Figure 1a, percolation AgNW random networks were coated on various substrates for purposes, such as Si wafer, glass, and polyethylene terephthalate (PET). Next, CVD-synthesized graphene was transferred onto AgNW random networks. Also, nanostructured hybrid films were formed in reverse order. Figure 1b presents a photograph of the fabricated transparent conductive films based on graphene-AgNW hybrid structures. The AgNW networks were covered by a polycrystalline graphene layer as displayed in Figure 1c. Here, we presented the SEM image of partially torn graphene in order to differentiate between the AgNW networks and graphene layer. Additionally, the graphene as a top layer was colored to provide a clear distinction between materials. Also, an AFM image of graphene-AgNW hybrid film exhibits the percolated AgNW networks on the graphene layer, as illustrated in Figure 1d. Lower spin rates lead to smaller standard deviations of *R*_*s*_ than higher spin rates (Figure 2). As expected, the *R*_*s*_ values of both films are reduced with decreasing spin coating rates. The transmittance value of the hybrid films was 2% to 3% less than that of AgNW networks due to the absorbance of monolayer graphene (2.3% of visible light) (Figure 2). This outstanding optical transparency of the hybrid films in visible light is comparable to that of ITO.

### Bendability of the hybrid structures

**Figure 3:**
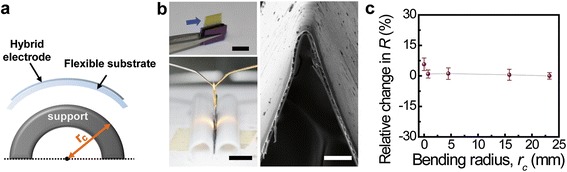
**Flexibility of the graphene-AgNW hybrid nanostructures: bending and folding. (a)** A schematic diagram of the bending process. **(b)** Photos (left) and a SEM image (right) of hybrid film folded in half. Black scale bars are 5 mm and white scale bar is 20 μm. **(c)** The dependence of relative change in the resistance as a function of bending radius.

Mechanical flexibility is another key factor of transparent electrodes for next-generation stretchable and wearable electronics. We achieved ultimate flexibility including bending and folding capability of the hybrid structures in Figure 3 [40]. For this study, a graphene layer was transferred onto a prepared 2-μm-thick polyimide substrate (see Experimental methods), followed by spin casting of AgNW dispersions. Figure 3a illustrates a schematic image for the bending test of the prepared hybrid electrodes. A flexible substrate with hybrid films was placed on cylindrical supports that had a diverse radius of curvature (*r*_*c*_), and this *r*_*c*_ can be estimated by measuring the outer diameter of the supports. In this manner, the hybrid samples were wrapped on different cylinder-shaped objects, such as a plastic straw and a glass capillary tube. For extreme bending, the hybrid films on the substrates were folded in half by pressing two pieces of Si wafer together using tape to attach both ends of the polyimide to the wafer (see left two photographs of Figure 3b). To estimate the *r*_*c*_ of the folded hybrid electrodes, a side view of the prepared sample was characterized by SEM. The resulting SEM image of the folded part is presented in the right inset of Figure 3b, and the *r*_*c*_ of this folding was calculated about 3.7 μm. Figure 3c shows the relative difference in *R* as a function of *r*_*c*_. This *x*-axis can also be re-interpreted as a bending-induced strain (*ε*) [10,12] according to *ε* = (*t*_*s*_ + *t*_*f*_)/2*r*_*c*_, where *t*_*s*_ and *t*_*f*_ are the thickness of the substrate and film, respectively. Although this folding leads to approximately 27% bending strain, a negligible change in *R* occurs for bending to radii of curvatures as small as 3.7 μm.

### Stretchability of the hybrid structures

**Figure 4:**
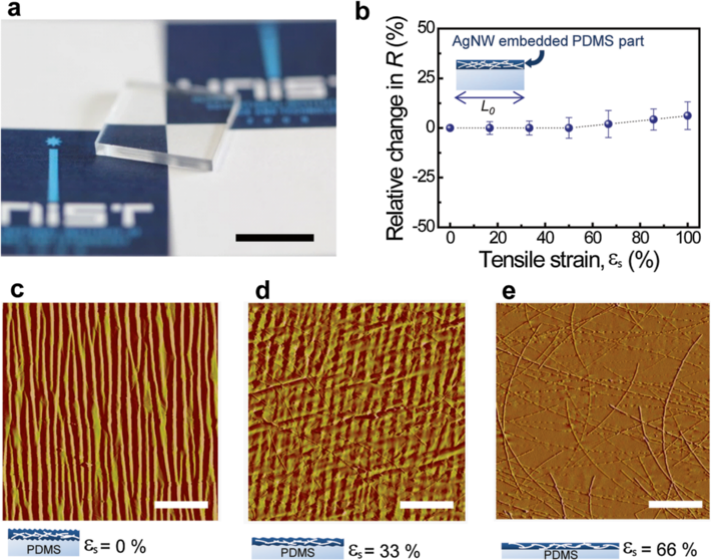
**Stretchability of graphene-AgNW-embedded hybrid electrodes. (a)** A photo of the hybrid electrode formed on a PDMS substrate. Scale bar is 1 cm. **(b)** Relative difference in the resistance as a function of tensile strain for hybrid structures embedded in PDMS. The AFM topographies of hybrid structures on PDMS **(c)** when releasing, **(d)** when re-stretching up to 33%, and **(e)** when re-stretching up to 66% after stretching up to 100%. (Here, AgNW networks were embedded in PDMS and a graphene layer was transferred onto the AgNW-embedded PDMS). Scale bars are 5 μm.

In addition to the extreme folding, the excellent elasticity of graphene [41] and the mesh structures of AgNW random networks [22,27,40,42] enable the hybrid electrodes to be mechanically stretchable without loss of electrical conductance. In order to improve stretchability, many researcher groups have studied geometric structuring such as formation of serpentine or horseshoe patterns, or buckling [43-45]. These strategies can be applied to a wide variety of materials, but these cause the loss of space and nonplanar structures due to tilting or buckling. In our approach, graphene-AgNWs hybrid structures were used to alleviate aforementioned limitations. The stretching characteristics of conducting nanomaterials embedded elastomeric substrates have been recently investigated [46-48]. For the investigation of the stretchable characteristics of the hybrid structures, a polydimethylsiloxane (PDMS) elastomeric substrate was used instead of the polyimide. Figure 4a illustrates a photo of the hybrid structures on PDMS, which is rested on a piece of paper with a logo to demonstrate its good transparent properties. For this study, AgNW networks were formed by using two kinds of methods, and the first experiment was carried out using AgNWs embedded in PDMS, followed by transfer as an as-synthesized graphene layer onto the AgNW networks. After clamping one of the prepared samples with two fixtures connected to the current–voltage measurement system, it was stretched to specific elongation lengths using a mechanical apparatus. Firstly, the AgNW-embedded hybrid sample was tested, and the relative difference in the *R* under various tensile strains (σ) was plotted in Figure 4b. Here, the tensile strain is the ratio of extension to original length. This graphene-AgNW (embedded) hybrid electrode can be stretched up to 100% tensile strain with negligible resistance change (Figure 4b), and then it was released. Although the local areas of the graphene can be torn by this stretching, AgNWs bridge gaps of the torn graphene and hence electrons can still pass through the nanowires across the cracked graphene. A schematic (bottom inset) and AFM image of fully released hybrid electrode without external tensile strain (σ = 0%) are exhibited in Figure 4c, and the samples that were re-stretched up to 33% and 66% tensile strain are depicted in Figure 4d,e, respectively. As described in Figure 4c, a severely uneven and buckling surface was observed when the σ was 0%. When these hybrid structures on PDMS were stretched again, the wavelength and amplitude of the surface waves increased and decreased, respectively, as shown in Figure 4d,e. This buckling structure of the surface is attributed to the friction force between the embedded AgNWs and the PDMS during repeated stretching and releasing tests [49].

**Figure 5:**
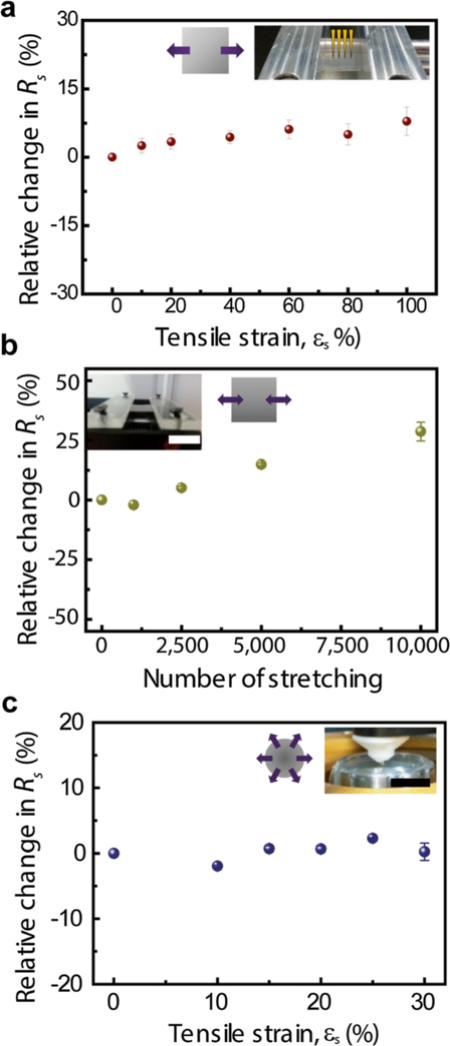
**Uniaxial and multi-axial stretchability of the hybrid structures. (a)** The relative difference in the resistance of fabricated hybrid films on PDMS as a function of tensile strain toward uniaxial direction. (Here, AgNW networks were spin-coated on PDMS and a graphene layer was transferred onto the AgNW-coated PDMS.) **(b)** A graph for 10,000 times cyclic fatigue test of the hybrid film. Scale bar is 1 cm. **(c)** Relative change in the sheet resistance of hybrid nanostructures on PDMS according to tensile strain toward multi-axial direction. Scale bar is 1 cm.

As the second form of hybrid sample, the AgNWs film was formed directly on PDMS using spin coating followed by transfer as an as-synthesized graphene layer onto the AgNW networks. The stretching testing was also done to examine the stretchable properties by elongating toward the uniaxial and multi-axial direction for the graphene-AgNW (spin-coated) hybrid electrode. Here, a multi-axial direction means the centrifugal force direction parallel with the plane of a fabricated hybrid films. The biaxial direction stretching of film has been reported [43] but that does not mean the centrifugal force direction stretching. After this hybrid sample was fixed to a mechanical apparatus, it was stretched toward the uniaxial direction and its *R*_*s*_ values were measured at specific stretching strains. Figure 5a shows the relative change in *R*_*s*_ according to increase of *ε*, and this graphene-AgNW (spin-coated) hybrid electrode can also be stretched up to 100% tensile strain without significant change. To investigate the durability against consecutive stretching and releasing, the *R*_*s*_ of the sample was measured during cyclic stretching tests (10,000 cycles at up to 100% tensile strain with a rate of 2.54 mm s^−1^) and it was almost constant without notable deformation, as plotted in Figure 5b. To confirm its further performance as a stretchable electrode, we assessed the *R*_*s*_ of our hybrid sample during stretching toward the multi-axial direction at up to 30% by using a homemade cylinder-shaped stretching stage, and the relative difference *R*_*s*_ was recorded at less than 5% (Figure 5c). Here, the maximum tensile strain was 30% due to the mechanical limitation of the stretching equipment. The mechanical stability of our hybrid nanostructures against bending (including extreme folding) and stretching is superior compared to ITO, which can be cracked by applying bending or tensile strain of approximately 1%.

### The effect of pattern size on electrical properties

**Figure 6:**
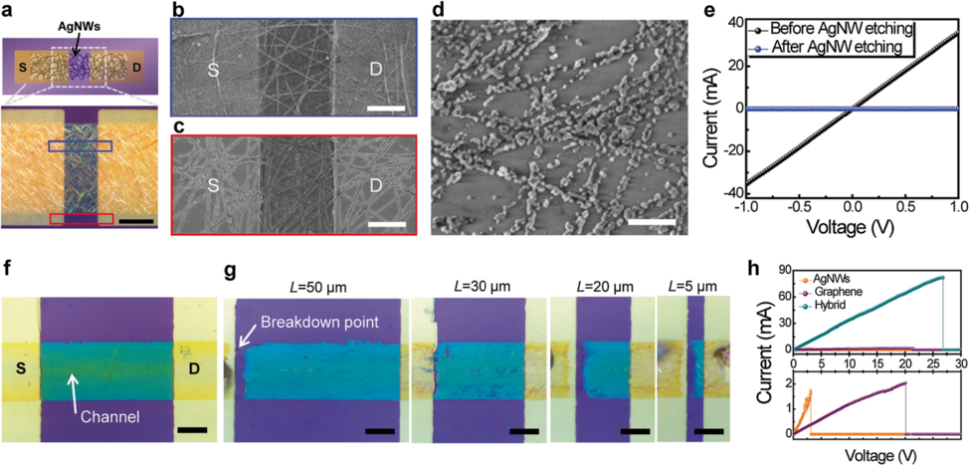
**Patterning of AgNW networks and breakdown characteristics of the hybrid pattern. (a)** An optical microscope image of patterned AgNW networks as channel by using dry etching. Top inset of (a) A schematic diagram of a device layout with patterned AgNW networks as channel. Scale bar is 30 μm. SEM images of AgNW networks **(b)** unexposed and **(c)** exposed to oxygen plasma. Scale bars are 5 μm. **(d)** A magnified SEM image of disconnected AgNW networks after dry etching process. Scale bar is 500 nm. **(e)** Comparison of electrical conductance of the AgNWs before and after reactive ion etching. **(f)** An optical microscopic image of patterned graphene-AgNW hybrid films by using dry and wet etching process. Scale bar is 10 μm. **(g)** Optical images of patterned hybrid films with various channel length after electrical breakdown. Scale bars are 10 μm. **(h)** *I-V* characteristics of AgNWs, graphene, and their hybrid channels (channel width: 10 μm, length: 50 μm). The lower graph is the magnification of the upper graph.

The electrical conductance of the nanostructured films can be changed by pattern size, such as via the widths and lengths of a pattern [40]. For this study, the fabrication process began with the evaporation of metal contact pads of Cr/Au (2/300 nm) onto a 300-nm-thick SiO_2_ on Si wafer, and then graphene, AgNW networks, and their hybrid structures were formed on this substrate with metal pads, respectively. The patterning of the aforementioned three materials with various widths and lengths was performed by using photolithography and dry and wet etching processes, followed by measurement and comparison of their *R* (see Experimental methods). Figure 6a shows an optical microscope image of the patterned AgNW random networks with a channel length and width of 30 and 70 μm, respectively. The top inset of Figure 6a exhibits a schematic illustration of a device layout with patterned AgNW networks as a channel. Figure 6b,c presents SEM images of the AgNW networks which were unexposed and exposed to oxygen plasma, respectively. Here, it is important to note that AgNWs remained with slightly modified shapes after oxygen plasma exposure (red rectangular in Figure 6a,c). To inspect in detail, the magnified SEM image of AgNW networks affected from oxygen plasma was used as described in Figure 6d, and this SEM image presented disconnected, fattened, and oxidized AgNW residues after reactive ion etching (RIE). Figure 6e presents the electrical conductance of AgNW networks before and after exposure to oxygen plasma. As expected, the AgNW networks were electrically nonconductive after the RIE process. From these results, we confirmed that locally nonconductive areas of the AgNW network films can be made by using photolithographical patterning and a RIE process.

In addition, the robustness of conductive materials that can withstand electrical load becomes an important feature for next-generation electronics, as devices are highly integrated and miniaturized. This is because the electric field is inversely proportional to the length of pattern. To assess and compare the robustness of nanostructured films against electrical loads, patterned samples of graphene, AgNWs, and their hybrid structures as channels with a width of 10 and 20 μm were prepared on a metal electrode deposited onto a Si wafer (with 300-nm-thick SiO_2_). Figure 6f shows an optical microscope image for one of the fabricated devices with the graphene-AgNW hybrid structures as a channel (blue area in Figure 6f) and Cr/Au (2 nm/300 nm) as S/D electrodes (yellow area in Figure 6f). Here, the width and length of hybrid channel are 20 and 50 μm, respectively. To compare the condition of channel materials before and after electrical breakdown, we presented the optical images of prepared devices with the hybrid channel with lengths that varied from 5 to 50 μm after the breakdown test in Figure 6g. In contrast to Figure 6f, all the channels with different lengths in Figure 6g were disconnected at the left end of the channels. In other words, the electrical breakdown occurred near one of the contact points between the hybrid channel and metal electrodes. The breakdown phenomena of hybrid structures are opposite to those of graphene where the breakdown point is nearly at the center of the channel due to Joule heating and are similar to those of AgNW. To inspect the electrical breakdown of the three nanomaterials, the *I-V* characteristics of graphene, AgNWs, and the hybrid structures with various channel lengths (*L*) were measured in an ambient condition. As one of the results, the top graph of Figure 6h describes breakdown behaviors of the three materials, when the length and width of channels were at 50 and 10 μm, respectively. In order to clearly distinguish the results between the three kinds of materials, magnified *y*-axis graphs are presented together at the bottom of Figure 6h. The slopes in the *I*-*V* curves indicate electrical conductance, and the narrow pattern size (channel length = 10 μm) caused the relatively high resistance of AgNW networks, compared to the resistance of the hybrid structures, because only small portions of the conductive pathways along the NWs remained inside the patterns. For all the three cases, currents increase almost linearly with the applied voltage before their breakdown. Based on these results, we compared breakdown bias (*V*_breakdown_) according to various *L* of these three different materials. In the case of AgNW networks that can be degraded by electromigration [50], electrical breakdown occurs at a relatively low voltage (*V*_breakdown_ < approximately 4 V) for a channel length of approximately ≥30 μm (average length of AgNWs) due to the large aspect ratio of NWs and NW-NW contact resistances. On the other hand, the *V*_breakdown_ of graphene is higher than that of AgNW networks. This polycrystalline graphene can be damaged by Joule heating and oxidation at the defect sites [51], and *V*_breakdown_ of graphene also decreases when the channel length falls below the threshold related to the grain sizes. In the case of graphene-AgNW hybrid nanostructures, the graphene underneath AgNWs can create a path to dissipate heat and electrical stress, which can make AgNWs sustainable against breakdown at relatively high electric bias. Therefore, the hybrid structures exhibit the highest values of *V*_breakdown_ among the three materials (graphene, AgNW networks, and the hybrid). Also, the hybrid electrode can flow the highest current against its breakdown due to its low resistance. The hybridization of the graphene and AgNWs give the hybrid structures strong endurance against electrical load, so this improved robustness is another advantage of the hybrid electrode.

## Conclusions

The results presented here demonstrate the superior characteristics of graphene-AgNW hybrid nanostructures as flexible, stretchable, and transparent electrodes. From the fundamental study, we confirmed that the hybridization between AgNWs and CVD synthesized graphene could significantly enhance electrical properties such as low *R*_*s*_ with negligible degradation of optical transparency and has strong robustness against electrical breakdown. In the reliability test, the hybrid structures preserve their outstanding properties against thermal oxidation due to a graphene passivation layer covering AgNW networks. Furthermore, our hybrid electrode presents superb mechanical flexibility and stretchability such as complete folding in half (27% in bending strain), 10,000 times in stretching cycles with 100% tensile strain toward the uniaxial direction, and 30% stretching in tensile strain toward the multi-axial direction. We believe that the hybridization with two-dimensional and one-dimensional nanomaterials presents a promising strategy toward stretchable, wearable electronics and implantable biosensor devices.
